# Supplementary material for: Pediatric Anesthesia Providers’ Perspective on the Real-Life Implementation of the Philips Visual Patient Avatar: A Qualitative Study
Source: Children (Basel). 2023 Nov 24;10(12):1841. doi: 10.3390/children10121841 (PMC10741887; doi:10.3390/children10121841)
Supplement: Supplementary file 1 [file children-10-01841-s001.zip › File S2 Interview guide.pdf]

Interview Guide

Part 1: Demographic characteristics

1. Please enter your age: ……….
2. Please indicate gender:<br>a) Female;<br>b) Male;<br>c) Other gender identity: ……….
3. Please indicate your role:<br>a) Certified nurse anesthetist;<br>b) Resident 1-2 years of training;<br>c) Resident 3-5 years of training;<br>d) Resident with > 5 years of experience;<br>e) Staff anesthesiologist.
4. Please indicate your experience in anesthesia (in years): ……….
5. Have you reviewed any Visual Patient educational material? If so, which ones?<br>a) Visual Patient Avatar short user guide (Intranet);<br>b) Visual Patient Avatar educational video (Intranet);<br>c) Both;<br>d) None.

Part 2: Open questions about Visual Patient

1. Having worked with Visual Patient at the University Hospital Zurich, what special aspects of Visual Patient can you imagine in the context of pediatric anesthesia? (Please list possible advantages and disadvantages).
2. Do you think the children/parents would like Visual Patient (e.g. how might they react to it)?

Translated with www.DeepL.com/Translator (free version)
