# Supplementary material for: Pediatric Anesthesia Providers’ Perspective on the Real-Life Implementation of the Philips Visual Patient Avatar: A Qualitative Study
Source: Children (Basel). 2023 Nov 24;10(12):1841. doi: 10.3390/children10121841 (PMC10741887; doi:10.3390/children10121841)
Supplement: Supplementary file 1 [file children-10-01841-s001.zip › File S3 Translated interview answers.pdf]

**Participant 1**

***After having worked with Visual Patient, what special considerations can you make about Visual Patient in pediatric anesthesia?***
***(List possible benefits and potential for improvement.)***

+ Well-displayed temperature.
- Alarm thresholds need to be adapted to children of different ages.

***Do you think the children/parents would like Visual Patient?***
***(How might they react to it?)***

- Children: Children like Visual Patient: its sympathetic appearance, friendly impression.
- Parents: Explanation of different parameters and their meaning may be necessary.

**Participant 2**

***After having worked with Visual Patient, what special considerations can you make about Visual Patient in pediatric anesthesia?***
***(List possible benefits and potential for improvement.)***

+ Even faster problem recognition, especially when the child`s temperature or oxygen saturation drops.
- Alarm threshold adaptation for children of different ages is needed.

***Do you think the children/parents would like Visual Patient?***
***(How might they react to it?)***

- Children: Visual Patient offers the possibility of entertaining children during certain procedures.
- Children: Visual Patient may need an adjustment of the awake face, as it tends to trigger fear.

**Participant 3**

***After having worked with Visual Patient, what special considerations can you make about Visual Patient in pediatric anesthesia?***
***(List possible benefits and potential for improvement.)***

+ From the position on the patient`s head, e.g., during anesthesia induction (far from the monitor), changes in the vital parameters can be quickly recognized.

- The very high pediatric heart rate is visually distracting in Visual Patient.

***Do you think the children/parents would like Visual Patient?***
***(How might they react to it?)***

- Children: Children could be interested.
- Parents: I experience it in Caesarean operations: the patient and the partner react very positively to Visual Patient; so I imagine that parents in pediatric anesthesia setting would also respond positively.

**Participant 4**

***After having worked with Visual Patient, what special considerations can you make about Visual Patient in pediatric anesthesia?***
***(List possible benefits and potential for improvement.)***

\+ Better temperature control.

\- Not yet adapted to children's vital signs.

\- Visual Patient can distract care providers from the patient - as the clinical assessment of the child itself is more important.

***Do you think the children/parents would like Visual Patient?***
***(How might they react to it?)***

- Children and Parents: I think both – children and parents – would like Visual Patient very much.

**Participant 5**

***After having worked with Visual Patient, what special considerations can you make about Visual Patient in pediatric anesthesia?***
***(List possible benefits and potential for improvement.)***

+ Good visualization of the changes in body temperature.

- Visualization of high heart rates makes Visual Patient appear too "nervous".

***Do you think the children/parents would like Visual Patient?***
***(How might they react to it?)***

- Children: Yes, in the sense of entertainment.

**Participant 6**

***After having worked with Visual Patient, what special considerations can you make about Visual Patient in pediatric anesthesia?***
***(List possible benefits and potential for improvement.)***

+ You can immediately see that something is wrong.

***Do you think the children/parents would like Visual Patient?***
***(How might they react to it?)***

- Children: Children definitely benefit from Visual Patient: you can explain the process to them better, and it also has an entertainment value.

**Participant 7**

***After having worked with Visual Patient, what special considerations can you make about Visual Patient in pediatric anesthesia?***
***(List possible benefits and potential for improvement.)***

+ Additional visual information about hypoxia + body temperature - ``quicker`` visual information.

- In a critical situation, I would not pay much attention to visualizations. You need numbers for that.

***Do you think the children/parents would like Visual Patient?***
***(How might they react to it?)***

- Children: they would like it because it looks funny. It could help with distraction, but a bit far away for that.

- Parents: probably also positive. With anxious parents, however, it could also lead to insecurity (thresholds often lead to changes in the VP when a care provider is still satisfied with the values, e.g., BIS 55 → Eyes open.

**Participant 8**

***After having worked with Visual Patient, what special considerations can you make about Visual Patient in pediatric anesthesia?***
***(List possible benefits and potential for improvement.)***

-

***Do you think the children/parents would like Visual Patient?***
***(How might they react to it?)***

- Children: I think children would love to look at Visual Patient.
- Parents: For me, this is absolutely unsuitable for parents, because parents cannot check facts and this increases insecurity and fear for the child.

**Participant 9**

***After having worked with Visual Patient, what special considerations can you make about Visual Patient in pediatric anesthesia?***
***(List possible benefits and potential for improvement.)***

+ Quick overview in critical situations.

+ Good for situation awareness just when, e.g., a lot of attention is absorbed with the distraction of the child at the beginning of the induction, a good picture of the overall situation can be formed by quickly looking at Visual Patient or the focus is drawn to the problem, if there is one.

+ Good tool to distract the child, exciting to look at, attracts attention through flashing and colors.

+ Good way to explain certain monitoring (or even individual parameters) to the child.

- There should be more profiles of VPs, each for different age groups in pediatric anesthesia because with the standard pediatric profile, the alarms do not always fit with very small children or even older children, and it then alarms wrongly and distracts.

***Do you think the children/parents would like Visual Patient?***
***(How might they react to it?)***

- Children: Children react very positively to VP: curious, find it exciting or even funny.
- Parents: Parents might be irritated, possibly worried, if everything flashes quickly or the VP turns purple.

**Participant 10**

***After having worked with Visual Patient, what special considerations can you make about Visual Patient in pediatric anesthesia?***
***(List possible benefits and potential for improvement.)***

- Currently unsuitable for young children as the thresholds cannot be adjusted.

***Do you think the children/parents would like Visual Patient?***
***(How might they react to it?)***

- Children: Children would probably be excited and parents too.

**Participant 11**

***After having worked with Visual Patient, what special considerations can you make about Visual Patient in pediatric anesthesia?***
***(List possible benefits and potential for improvement.)***

+ The visual temperature representation is useful.
- As long as Visual Patient does not adopt the thresholds, the application is rather irritating for children. In order to improve this, thresholds must be adjustable for each age.

***Do you think the children/parents would like Visual Patient?***
***(How might they react to it?)***

- Children: The children would like Visual Patient.
- Parents: With parents, I could be distressing when the colors change without you knowing what it means.

**Participant 12**

***After having worked with Visual Patient, what special considerations can you make about Visual Patient in pediatric anesthesia?***
***(List possible benefits and potential for improvement.)***

- Very different normal values depending on age. It should be adapted for different children.

***Do you think the children/parents would like Visual Patient?***
***(How might they react to it?)***

- Children: Visual Patient could be made a little friendlier for children (smile?)
- Parents: Parents perceive negative changes in Visual Patient more than changes in the normal curves.
- Children and parents: Overall, positive reaction from children and parents.

**Participant 13**

***After having worked with Visual Patient, what special considerations can you make about Visual Patient in pediatric anesthesia?***
***(List possible benefits and potential for improvement.)***

+ Visualization in general.
- Visualization of high pediatric heart rate can cause stress.

***Do you think the children/parents would like Visual Patient?***
***(How might they react to it?)***

- Children: Children would be interested.
- Parents: Parents rather positive.

**Participant 14**

***After having worked with Visual Patient, what special considerations can you make about Visual Patient in pediatric anesthesia?***
***(List possible benefits and potential for improvement.)***

\+ Possibly quicker recognition of the problems through good visualizations.

\- Visualization of heart rate confuses.

***Do you think the children/parents would like Visual Patient?***
***(How might they react to it?)***

- Children: Neutral
- Parents: Also neutral, because they do not see it → you only see it in the operating theater.

Translated with www.DeepL.com/Translator (free version)
